# PRINCIPLES OF QUEER GERONTOLOGY

**DOI:** 10.1093/geroni/igae098.1947

**Published:** 2024-12-31

**Authors:** Vanessa Fabbre, Austin Oswald, Sarah Jen

**Affiliations:** Washington University in St. Louis, St. Louis, Missouri, United States; Dalhousie University, Nova Scotia, Canada; University of Kansas, Lawrence, Kansas, United States

## Abstract

Gerontology has advanced greatly in recent decades in its attention to the lives of LGBTQ+ people. To this end, an increasing number of scholars have argued for a queering of gerontologists’ understanding of the human life course, as well as a queering of the field of gerontology itself. Yet, to date there exists no clear articulation, in practical terms, of what queer gerontology is and how it could be applied to scholarship and activism. Coming from a first-person, reflexive position, we argue that a clear vision of queer gerontology should be based on scholars’ historical use of queer theory to challenge normative ideas about the self and social life. In this presentation we will briefly present the historical landscape of LGBTQ+ aging studies, with an emphasis on recent conceptual and methodological advances, followed by a description of the iterative process through which we (as both early and mid-career scholars) developed the following five principles of queer gerontology: 1) Deeping Visibility and Awareness, whereby the identities and lives of LGBTQ+ and all oppressed peoples are elevated and illuminated; 2) Developing and Applying Critical Perspectives, whereby concepts and theories that challenge the status quo are engaged; 3) Challenging Oppression and Injustice, whereby critical theories are explicitly used to guide social change efforts; 4) Engaging Multiple Epistemologies and Modes of Inquiry, whereby scholars actively promote and use various approaches to scientific and social inquiry; and 5) Fostering Transdisciplinary Advancement, whereby scholars seek out and develop relationships that transcend traditional disciplinary boundaries.

